# The association between men’s family planning networks and contraceptive use among their female partners: an egocentric network study in Madagascar

**DOI:** 10.1186/s12889-021-10180-6

**Published:** 2021-01-25

**Authors:** Alison B. Comfort, Cynthia C. Harper, Alexander C. Tsai, Jessica M. Perkins, James Moody, Justin Ranjalahy Rasolofomana, Cora Alperin, Margaret Schultz, Anja Noeliarivelo Ranjalahy, Ravo Heriniaina, Paul J. Krezanoski

**Affiliations:** 1 University of California San Francisco, 550 16th Street, San Francisco, CA 94158 USA; 2 Opportunity Solutions International, San Francisco, CA USA; 3 Massachusetts General Hospital, Harvard Medical School, 125 Nashua Street, Suite 722, Boston, MA 02114 USA; 4 Peabody College of Education and Human Development, Vanderbilt University, PMB 90, 230 Appleton Place, Nashville, TN 37203-5721 USA; 5 Duke University, 268 Soc/Psych Building, Durham, NC 27708-0088 USA; 6 Institut National de Santé Publique et Communautaire, Ex-Ecole de Médecine de Befelatanana, BP 176 Antananarivo, Madagascar; 7 University of Chicago, 5438 S Woodlawn Avenue, Chicago, IL 60615 USA; 8 Institute for Global Health Sciences, University of California San Francisco, 550 16th Street, Third Floor, San Francisco, CA 94158 USA; 9 TANDEM SARL, Lot H 107, Merimanjaka, 102 Antananarivo, Madagascar; 10 Opportunity Solutions International, Lot III G17, Ambalavao, Madagascar; 11 University of California San Francisco, Zuckerberg San Francisco General Hospital, 1001 Potrero Ave, San Francisco, CA 94110 USA

**Keywords:** Social networks, Contraceptives, Family planning, Male partner, Influencers, Health providers, Community health workers, Reproductive health, Madagascar, Sub-Saharan Africa

## Abstract

**Background:**

Ensuring women have information, support and access to family planning (FP) services will allow women to exercise their reproductive autonomy and reduce maternal mortality, which remains high in countries such as Madagascar. Research shows that women’s social networks - their ties with partners, family members, friends, and providers - affect their contraceptive use. Few studies have considered the role of men’s social networks on women’s contraceptive use. Insofar as women’s contraceptive use may be influenced by their male partners, women’s contraceptive use may also be affected by their partner’s social networks. Men may differ by the types of ties they rely on for information and advice about FP. It is unknown whether differences in the composition of men’s FP networks matter for couples’ contraceptive use. This study assessed the association between men’s FP networks and couples’ contraceptive use.

**Methods:**

This egocentric network study was conducted among married/partnered men (*n* = 178) in rural Madagascar. Study participants listed who they relied on for FP information and advice, including health providers and social ties. They provided ties’ gender, age, relationship, and perceived support of contraceptive use. The primary outcome was couples’ contraceptive use, and explanatory variables included FP networks and their composition (no FP network, social-only network, provider-only network, and mixed network of social and provider ties). Analyses used generalized linear models specifying a Poisson distribution, with covariate adjustment and cluster robust standard errors.

**Results:**

Men who had FP networks were 1.9 times more likely to use modern contraception as a couple compared to men with no FP network (95% confidence interval [CI] 1.64–2.52; *p* ≤ 0.001). Compared to men with no FP network, men were more likely to use modern contraception if they had a social-only network, relative risk (RR) = 2.10 (95% CI, 1.65–2.68; p ≤ 0.001); a provider-only network, RR = 1.80 (95% CI, 1.54–2.11; *p* ≤ 0.001); or a mixed network, RR = 2.35 (95% CI, 1.97–2.80; *p* ≤ 0.001).

**Conclusions:**

Whether men have a FP network, be it provider or social ties, distinguishes if couples are using contraception. Interventions should focus on reaching men not only through providers but also through their social ties to foster communication and support for contraceptive use.

## Background

Ensuring universal access to family planning (FP) services is a priority in the 2030 Sustainable Development Goals [1]. Removing barriers to access FP services, as well as making sure women have the information and support they need to seek reproductive health services, receive respectful care, and be able to effectively use contraception when desired will allow women to exercise their reproductive autonomy [2]. Meeting women’s FP needs has been shown to help reduce maternal mortality, which is particularly high in sub-Saharan African countries such as Madagascar [3, 4]. While there is growing research showing that women’s social networks, that is their ties with family members, friends, and health care providers and the nature of these ties, affects their contraceptive use [5–14], male partners can also influence whether women use contraception [15]. Studies have shown that perceptions of whether male partners are supportive of contraceptive use can influence whether women use contraception [16–22]. For example, concern about opposition to contraceptive use, mainly from male partners, was identified as a barrier to contraceptive use among women in Madagascar [23]. Additional research has found that involving men in contraceptive counseling was associated with increased contraceptive use [24–27], though other studies have found negative or no effects [28, 29]. Most research on the role of men in contraceptive decision-making has focused on their individual characteristics, such as age, education, urbanicity, as well as social and cultural factors such as religious beliefs, desired family size, gender roles and male identity [30–34]. However, very few studies have moved beyond looking at men’s individual characteristics to consider whether men’s social networks influence women’s contraceptive use.

Insofar as women’s contraceptive use may be influenced by their male partners, women’s contraceptive use may also be influenced by the networks of their male partners, i.e., the ties that men have with their partner, family members, friends and health providers [35, 36]. Based on the nature of these ties, networks can provide informational, emotional, and financial support, offer opportunities for peer learning, and/or social engagement, while being influenced by the socio-cultural context within which they exist. Whereas researchers have increasingly used social network theory and analysis to explore how women’s reproductive health decisions are tied to others in their networks [37–39], very little research exists examining how the composition of men’s social networks may influence, in part, female partners’ contraceptive use. A longitudinal network study in Kenya found that couples were significantly more likely to use contraception if the male partner had at least 1 contraceptive user in their own network, adjusting for unmeasured confounding and the tendency for individuals to have ties to other individuals with similar characteristics (i.e., homophily) [9]. Another longitudinal study found that men in Ghana who received positive encouragement from their social network to use contraception were more likely to report using contraception with their partners [40].

Men may differ in the types of social ties they rely on for seeking information and advice about FP including contraceptive use. Some men may rely on health providers (e.g. community health workers [CHWs], doctors, nurses, or mid-wives) for FP and contraceptive information and advice, while others may rely on social ties, such as friends, partners, and family members. Men may also rely on a combination of both of these, or on no one at all. In-depth interviews among married men in Uganda found that men preferred to rely on partners for contraceptive information as opposed to health providers, peers, or mass media [41]. Focus group discussions with men in Kenya revealed that men did not trust either their health providers or their partners for FP information [33]. The extent to which the type and composition of ties in men’s FP networks impacts contraceptive use among couples is unknown.

While relying on health providers for advice is expected to be positively associated with couple’s contraceptive use, the influence of other kinds of ties within men’s social networks on couples’ contraceptive use is more ambiguous. A study among women in the United States showed that women tend to hear negative or incorrect information about contraception from their social relations compared to providers, but it is unclear if this might be the case for men as well [42]. However, this association has not been studies among men, especially within a resource-limited setting. Additionally, networks can constrain individual autonomy in reproductive health decisions by inducing individuals to align their behaviors with other network members [43, 44]. Information about the extent to which the composition of men’s networks for FP information and advice may be associated with couple’s contraceptive use is limited. Addressing this gap in the literature could inform the design of FP interventions that leverage men’s social networks to foster their engagement in contraceptive decision-making, support their partner’s choices in relation to contraception use and facilitate the goal of universal access to FP for all women.

In this egocentric network study among men in Madagascar, we collected data from men about who they turn to for information and advice regarding FP and how these FP ties are associated with their female partners’ contraceptive use. We assessed 1) whether men were more likely to report that their partner was using modern contraception if they personally had at least one FP tie to turn to for information and advice about FP, 2) whether the composition of men’s networks was differentially associated with couples’ contraceptive use (that is networks made up of social connections versus health care providers), 3) whether men who knew other individuals using contraception were more likely to report that their partner was using modern contraception, and 4) whether the characteristics of men’s ties, such as type of interpersonal relationship and the perceived support of contraceptive use by that person, was associated with couples’ contraceptive use.

## Methods

### Study participants and design

The study sample was recruited from 27 villages randomly selected from the 80 villages within a 5 km radius of the semi-urban town of Ambalavao in Fianarantsoa Province, Madagascar. Data collectors enumerated all households by village and then used random number draws to identify households for study participation. The total sample chosen from each village was proportionate to the number of households in the village, with a minimum of 5 households per village. Within each selected household, the data collector enumerated all men ages 18 years and older who were willing to answer questions about FP, and then randomly selected one man. Surveys were conducted in the local language (Malagasy) by male data collectors in order to facilitate study recruitment and elicit candid answers about reproductive health topics.

Prior to data collection, written informed consent was obtained, with verbal informed consent permitted for participants who could not read and write. All men who were randomly chosen within a household consented to participate, yielding a study sample of 208 men. Men who were not in partnership (that is, who were either divorced, widowed, or single; *n* = 17) and men whose partners were pregnant (*n* = 13) were excluded from this study. Thus, the final analytical sample included 178 study participants. Participants were provided with a small gift for their time and participation in the form of a *lamba,* a cloth used locally for multiple purposes. The study received approval from the National Ethics Committee in Madagascar and the University of California San Francisco Institutional Review Board. Cross-sectional data were collected from June to July 2019.

### Measures

The primary outcome was a binary measure of whether the participant reported that their partner is currently using modern contraception. We hereafter interpret this variable as the couple using modern contraception though it is understood that the measure is based on the male partner’s perception of his partner’s contraceptive use. We defined modern contraception to include injectables, the pill (oral contraceptives), implants, intra-uterine devices (IUD) and external condoms (i.e., male condoms). Participants were also asked if they knew anyone else using these modern methods, which was recorded as a dichotomous yes or no. Male and female sterilization were excluded as forms of modern contraception because of data validity issues. In our data, 15% of men reported using sterilization. For reference, in national surveys among reproductive age women in Madagascar, 4% report using female sterilization and 0.3% report that men were sterilized [45]. This discrepancy was likely due to a misunderstanding of the question, so we excluded reports of sterilization as a form of modern contraception.

Participants were also asked about their desire for pregnancy as a couple in the last 4 weeks (with answer categories for *wanted to become pregnant, did not want to, or was not certain*). Participants also reported who, within the couple, made decisions about contraceptive use (*participant, his partner, or together as a couple*). The number of births that the female partner had experienced was also elicited from participants. The number of births was used to reflect fertility, given high infant and child mortality in Madagascar [46]. Other data recorded included age, whether the participant was married and/or living with the partner or has partner but not living together, number of household members, whether attended school and highest grade attained, current occupation, estimated household earnings in the past month, and whether the household had electricity. Missingness for these variables is < 3% except for pregnancy desires (12%), contraceptive use decision-making (20%), and number of births (11%).

To collect network data, we adapted the people network survey developed by Brunson (2013) [47, 48] to ask about FP. We asked participants (egos) to list the individuals (alters) from whom they obtain information, advice, and/or guidance about FP.^1^ The question was worded as follows: “The purpose of these next questions is to gather accurate information about your people network; in other words, the people from whom you obtain information, advice and/or guidance about your use of family planning. Please take a moment to think about who these people might be. Please provide the first names of the people who have influenced your use of family planning.” Interviewers then prompted participants to review if they had forgotten any alters, prompting them to consider whether they wished to include their partner(s), siblings, parents, CHWs, and other health providers to ensure completeness. The maximum number of alters elicited from any study participant was 3. For each alter that was listed, name interpreter questions elicited alter age and gender, nature of the ego’s relationship with that alter, and the ego’s perception of that alter’s support of modern contraceptive use (categorized as supportive of modern contraceptive use vs. not supportive).

Several network measures were used as explanatory variables in separate analyses. The first explanatory variable was a dichotomous variable for whether the study participant named at least one individual in his FP network. The second was a categorical variable that included four mutually exclusive categories to represent network composition: 1) having no FP ties and thus no FP network, 2) all FP ties were composed of social relations only, including partner, other family members, and/or friends (hereafter referred to as *social-only FP network*); 3) all FP ties were composed of providers only, including CHWs, health educators, nurses, mid-wives or doctors (hereafter referred to as *provider-only FP network);* and 4) FP ties were composed of a combination of social and provider ties (hereafter referred to as a *mixed FP network*). The size of the FP network was categorized as 0 alters, 1 alter, or ≥ 2 alters.

There were no differences in contraceptive use or network composition by missingness of baseline covariates. If observations had missing covariates, then they were not included in the analysis. However, missingness of covariates was significant at *p* = 0.051 level, for differences in FP network composition. Men with no FP network were more likely to have missing covariates and therefore were not included in the analysis: 26% of men with missing covariates had no FP network compared to 16% of men with non-missing covariates.

### Analytical approach

We fitted a generalized linear model specifying a Poisson distribution with robust standard errors, interpreting the estimated incidence rate ratios as relative risk ratios (RR) [49]. In all analyses, the individual-level characteristics included age, number of births by partner, living together status, primary school completion, and household earnings. To avoid model over-specification, other individual characteristics were not included because of limited variation (electricity, occupation as farmer). Household size was included instead of number of births because of higher missingness rates (results are similar when births is included instead). Models also included fixed effects by sub-districts (the Malagasy *Fokontany,* which represents groups of villages). Analyses were conducted at the ego-level using robust standard errors clustered by sub-district. Additional analyses were conducted at the alter-level only using data from men with a FP network to examine the association between alter characteristics (gender, age, perceived support for contraceptive use, and type of relationship (e.g. CHW) and couple’s use of contraception, with robust standard errors clustered by sub-district. Alternative specifications included adjusting for: 1) couples’ pregnancy desire and 2) couples’ contraceptive decision-making. These covariates were not included in the main model because of higher missingness rates. In other specifications, we explored: 3) including both network composition and knowledge of contraceptive users variables, and 4) excluding partners as sources of advice in the network since the outcome simultaneously measures partners’ use of contraception. We also conducted an e-value sensitivity analysis to estimate how large the relative risk ratio of an unobserved confounder would need to be associated with both contraceptive use and men’s social networks in order to completely explain the associations in the study [50, 51].

## Results

### Descriptive statistics of sample

The mean age of the men in the study was 31 years; 141 (79%) were married or living with their partner, while 37 (21%) were living apart from their partner (Table 1). The mean number of reported births by their partner was 2.3 and the median was 2.0 (interquartile range [IQR], 1.0–3.0). Mean household size was 4.8. Almost all the men had attended school, with 116 (65%) reporting that they had completed primary school. Almost all men (148 [97%]) reported that farming was their main occupation. Only 2 (1%) reported having electricity in their home. Average household monthly earnings were reported to be 29 USD (~ 1 USD per day).^2^

**Table 1:** Summary characteristics of study sample (*n* = 178)

	(Mean ± s.d.) / n (%)	
**Demographics**		
Age (years)	31 ± 8.42	
Number of births by partner	2.30 ± 1.92	
Marital status (%)		
Married or living with partner	141	(79)
Has partner but not living together	37	(21)
Household size	4.79 ± 2.02	
Attended school (%)	173	(97)
Highest grade attained	6.12 ± 3.25	
Completed primary education (%)	116	(65)
**Socioeconomics**		
Occupation as farmer (%)	148	(97)
Household earnings (USD) (last month)	29.00 ± 35.02	
Home has electricity (%)	2	(01)
**Couples’ pregnancy desires** (%)		
Wants to get pregnant	3	(2)
Does not want to get pregnant	4	(3)
Uncertain about whether want to become pregnant	150	(96)
**Partner Relationship**		
Decision-making about contraceptive use with partner		
Man decides	6	(4)
His partner decides	39	(27)
Couple decides together	98	(69)

Ambivalence about desire for pregnancy as a couple was very common, with 150 (96%) men reporting that they and/or their partners were uncertain about wanting to become pregnant. When asked about who in their relationship makes decision about contraception, 6 (4%) said that the decision was the man’s decision, 39 (27%) said that it was the woman’s decision, and 98 (69%) said that it was the couples’ decision together.

Two-thirds of participants reported their partner was using modern contraception (114 [66%]) (Table 2). The most common methods were injectables (54 [31%]), the pill (31 [18%]), and the implant (27 [15%]), with few using an IUD (1 [1%]) or the external condom (2 [1%]). Three-quarters of men (134) knew someone else using modern contraception. Specifically, 93 (53%) knew someone using injectables, 88 (51%) knew someone using the pill and 69 (39%) knew someone using the implant.

**Table 2:** Study outcomes and explanatory variables among study sample (*n* = 178)

***Outcomes***	(Mean ± s.d.) / n (%)	
**Couple’s contraceptive use (current)**		
Any modern method (%)	114	(66)
Injectable	54	(31)
Pill	31	(18)
Implant	27	(15)
Intra-uterine device	1	(1)
External condom	2	(1)
No modern method (%)	59	(34)
***Network explanatory variables***		
Has a FP network (%)	146	(82)
*Composition of FP network*		
Social-only FP network	46	(26)
Provider-only FP network	96	(54)
Mixed FP network (both social and provider ties)	4	(2)
No FP network	32	(18)
Size of FP network	0.96 ± 0.60	
0 alters	32	(18)
1 alter	125	(70)
2 alters	17	(10)
3 alters	4	(2)
*Knows someone using:*		
Modern contraceptive method (%)	134	(78)
Injectable	93	(53)
Pill	88	(51)
Implant	69	(39)
Intra-uterine device	9	(5)
External condom	5	(3)
*Alter characteristics (reported by ego) (n = 170)*		
Age (years)	42.8 ± 10.32	
Female (%)	144	(88)
Supportive of contraceptive use (%)	143	(86)
Relationship to ego (%)		
Community health worker	25	(15)
Health educator	63	(37)
Health provider (mid-wife, nurse, doctor)	31	(18)
Friend	8	(5)
Partner	20	(12)
Sibling	3	(2)
Mother	16	(9)
Aunt	0	(0)
Other	4	(2)

Most men reported having a FP network (146 [82%]) with an average network size of 0.96 alters (s.d. 0.60). Thirty-two (18%) had no alters, 125 (70%) had 1 alter, 17 (10%) had 2 alters, and 4 (2%) had 3 alters. The composition of the FP network varied: 46 men (26%) had a social-only FP network, 96 men (54%) had a provider-only FP network, and 4 men (2%) had a mixed FP network, while 32 men (18%) identified no FP network. Most men (86%) who have a FP network nominated only 1 alter. Among the 170 nominated alters, the mean age was 43 years, almost all were women (144 [88%]), and 86% (143) were perceived to be supportive of contraceptive use. Provider relationships between ego and alter were more prevalent than social relationships. Among the nominated alters, 37% were health educators, 18% were health providers (nurses, mid-wives, and doctors) and 15% were CHWs. Five percent of alters were friends, 12% were partners, 9% were mothers and 2% were siblings.

### Differences in couples’ contraceptive use by composition of FP network

**Fig. 1:**
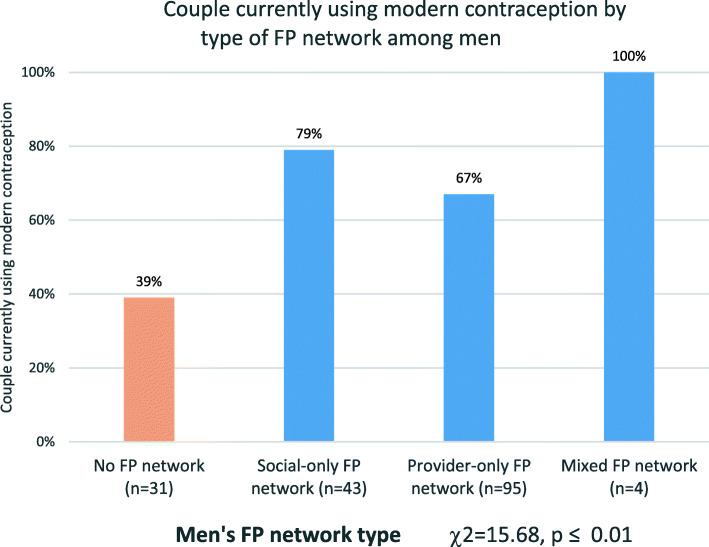
Couple currently using modern contraception by type of FP network among men

Chi-square testing indicated differences in contraceptive use by composition of the FP network (χ2 = 15.68, *p* ≤ 0.01) (Fig. 1). Among the 32 men with no FP network, 39% were using contraception. Among the 46 men with a social-only FP network, 79% were using contraception. Among the 96 men with a provider-only FP network, 67% were using contraception.

### Association between composition of FP network and couples’ use of contraception

Men who reported any kind of FP network were 1.9 times more likely to use modern contraception as a couple compared to men with no FP network (95% confidence interval [CI], 1.64–2.52; *p* ≤ 0.001), after adjusting for men’s individual characteristics (Table 3). Compared to men with no FP network, men who reported FP networks were more likely to report using modern contraception: having a social-only FP network, RR = 2.10 (95% CI, 1.65–2.68; p ≤ 0.001); having a provider-only FP network, RR = 1.80 (95% CI, 1.54–2.11; p ≤ 0.001); and a mixed FP network, RR = 2.35 (95% CI, 1.97–2.80; p ≤ 0.001). Reported contraceptive use was similar between men with a social-only FP network compared to men with a provider-only FP network (χ2 = 2.97, *p* = 0.09). However, men with a mixed network were more likely to use contraception compared to men with a provider-only network (χ2 = 8.41, *p* = 0.01). Compared to men with no FP network, men with one alter in their FP network were 2.0 times more likely to use contraception (95% CI, 1.68–2.35; p ≤ 0.001) and men with 2 or 3 alters in their FP network were 1.6 times more likely to use contraception (95% CI, 1.10–2.27; *p* ≤ 0.05). Among participants with a FP network, the size of the FP network was not differentially associated with contraceptive use (χ2 = 1.56, *p* = 0.21). Most individual-level characteristics were not significantly associated with couples’ contraceptive use. However, there were significant differences by sub-districts (Fokontanys).

**Table 3:** Association between men’s FP network characteristics and couples’ use of contraception using generalized linear model with Poisson regression and robust standard errors

	Dependent variable: Couple currently using modern contraception			
	RR	RR	RR	RR
*Independent variables*	(1)	(2)	(3)	(4)
Has a FP network	1.92^c^			
	(0.16)			
Composition of FP network				
No FP network *(Reference group)*		–		
Social-only FP network		2.10^c^		
		(0.26)		
Provider-only FP network		1.80^c^		
		(0.14)		
Mixed FP network		2.35^c^		
		(0.21)		
Size of FP network				
No FP network (*Reference group)*			–	
One alter in network			1.98^c^	
			(0.17)	
Two or more alters in network			1.58^a^	
			(0.29)	
Knows someone using modern contraceptive method				1.41^a^
				(0.21)
Age (years)	0.99	0.99	0.99^a^	1.00
	(0.01)	(0.01)	(0.01)	(0.00)
Household size	1.00	1.00	1.00	0.98
	(0.04)	(0.04)	(0.04)	(0.04)
Currently married	1.05	1.06	1.04	0.99
	(0.09)	(0.09)	(0.08)	(0.07)
Completed primary school education	0.98	0.99	0.98	0.96
	(0.08)	(0.09)	(0.08)	(0.07)
Monthly household earnings (USD)	1.00^b^	1.00^b^	1.00^b^	1.00^b^
	(0.00)	(0.00)	(0.00)	(0.00)
Sub-district 1 *(reference group)*	–	–	–	–
Sub-district 2	0.00^c^	0.00^c^	0.00^c^	0.00^c^
	(0.00)	(0.00)	(0.00)	(0.00)
Sub-district 3	0.00^c^	0.00^c^	0.00^c^	0.00^c^
	(0.00)	(0.00)	(0.00)	(0.00)
Sub-district 4	1.01	1.04	1.03	0.98
	(0.03)	(0.05)	(0.05)	(0.04)
Sub-district 5	0.94^a^	0.91^a^	0.99	0.94
	(0.03)	(0.03)	(0.04)	(0.05)
Sub-district 6	0.71^c^	0.73^c^	0.70^c^	0.74^c^
	(0.02)	(0.03)	(0.02)	(0.04)
Sub-district 7	0.96	0.93	0.98	0.92
	(0.03)	(0.04)	(0.04)	(0.04)
Sub-district 8	0.60^c^	0.60^c^	0.63^c^	0.51^c^
	(0.02)	(0.02)	(0.03)	(0.01)
Sub-district 9	0.73^c^	0.69^c^	0.76^c^	0.69^c^
	(0.02)	(0.04)	(0.03)	(0.01)
Sub-district 10	0.72^c^	0.71^c^	0.75^c^	0.68^c^
	(0.02)	(0.02)	(0.03)	(0.02)
Observations	164	164	164	159
Mean of dependent variable	0.65	0.65	0.65	0.64

^a^ significant at 0.05; ^b^ significant at 0.01; ^c^ significant at 0.001

Note: Each column presents the findings of a separate regression model. Covariates included in each of these 4 regression models were age, household size, marital status, primary school completion, and household earnings. We also included sub-district fixed effects. We used cluster robust standard errors at the sub-district level. Social-only FP network included social ties such as partner, friend, and other family members. Provider-only FP network included provider ties such as CHWs, health educators, nurses, mid-wives, and doctors. A mixed FP network included both social ties and providers ties. RR = Relative risk ratio

Men with an alter who was perceived to be supportive of contraception were more likely to use contraception (Table 4). The associations between contraceptive use and alter gender or age were not statistically significant. Compared with men who relied on doctors, nurses, and mid-wives (reference group), men were more likely to use contraception if they relied on their partner (RR = 1.3 [95% CI, 1.08–1.65; *p* ≤ 0.01]) or their sibling (RR = 1.5 [95% CI, 1.14–2.10; p ≤ 0.01]). The estimated relative risks for CHWs were positive but imprecisely estimated (*p*-value = 0.13). The association with other types of alter relationships was not statistically significant. In the second specification, being married was positively associated with contraceptive use, while completing primary school was negatively associated with contraceptive use. In all specifications, there were significant differences by sub-districts (Fokontanys).

**Table 4:** Association between alter characteristics and use of contraception using generalized linear model with Poisson regression and robust standard errors (alter-level analysis)

	Dependent variable: Couple currently using modern contraception		
	RR	RR	RR
*Alter characteristics*	(1)	(2)	(3)
Female	1.04		
	(0.26)		
Age (years)	0.99		
	(0.00)		
Supportive of contraceptive use		1.12e+ 07***	
		(5.30e+ 06)	
*Type of relation*			
Provider (nurse, mid-wife, doctor) *(Reference group)*			–
Community health worker			1.31
			(0.23)
Health educator			0.83
			(0.10)
Friend			1.22
			(0.25)
Partner			1.33**
			(0.14)
Mother/aunt			1.02
			(0.20)
Sibling			1.55**
			(0.24)
Other			0.76
			(0.32)
Age (years)	1.00	1.00	1.00
	(0.01)	(0.01)	(0.01)
Household size	1.00	0.96	0.98
	(0.03)	(0.02)	(0.03)
Currently married	1.11	1.21**	1.11
	(0.08)	(0.08)	(0.15)
Completed primary school education	0.82*	0.85***	0.91
	(0.08)	(0.03)	(0.14)
Monthly household earnings (USD)	1.00*	1.00	1.00
	(0.00)	(0.00)	(0.00)
Sub-district 1 *(reference group)*	–	–	–
Sub-district 3	0.00***	0.00***	0.00***
	(0.00)	(0.00)	(0.00)
Sub-district 4	0.96	0.94	1.02
	(0.08)	(0.04)	(0.07)
Sub-district 5	0.94	1.05	0.86
	(0.07)	(0.05)	(0.09)
Sub-district 6	0.71***	0.90	0.71***
	(0.07)	(0.05)	(0.05)
Sub-district 7	0.86*	0.99	0.76**
	(0.05)	(0.04)	(0.07)
Sub-district 8	0.54***	1.22***	0.60***
	(0.06)	(0.05)	(0.04)
Sub-district 9	0.58***	0.73**	0.38***
	(0.04)	(0.08)	(0.04)
Sub-district 10	0.71***	0.85**	0.65***
	(0.04)	(0.04)	(0.05)
Observations	143	156	159
Mean of dependent variable	0.69	0.71	0.69

Note: This analysis was restricted to men who had a FP network. Each column presents the findings of a separate regression model. Covariates included in each of these 4 regression models were age, household size, marital status, primary school completion, and household earnings. We also included sub-district fixed effects. Sub-district 2 is not included in this analysis due to missing data from the 5 respondents in that sub-district which had reported an alter but for whom specific alter characteristics are missing likely due to data collection error. We used cluster robust standard errors at the sub-district level. *RR* Relative risk ratio

Among the 37 men who did not know someone else using contraception, 18 (47%) were using contraception as a couple, whereas among the 131 men who did know someone else using contraception, 91 (69%) were using contraception (χ2 = 5.49, *p* ≤ 0.05). Regression analyses confirmed that, after adjusting for individual-level characteristics, men who knew someone else using contraception were 1.4 times more likely to use contraception (95% CI, 1.05–1.90; p ≤ 0.05).

### Sensitivity analyses

The results were consistent after adjusting for additional covariates, including pregnancy desires as a couple. Compared to men in couples who wanted to become pregnant (reference group), men in couples who wanted to avoid pregnancy were significantly more likely to use contraception (Appendix A). Similarly, men in couples who were uncertain about wanting to become pregnant were also significantly more likely to use contraception. When covariates for couples’ decision-making about contraception were included, all coefficients on type of FP network (social-only, provider-only, and mixed FP network) were significant. There was no association between who makes contraceptive decisions and contraceptive use. While the main analyses showed that individual-level characteristics were not associated with contraceptive use, omitting these variables yielded similar results though the magnitude of the coefficients of interest were slightly smaller suggesting that it was important to adjust for these variables. When both the measure for network composition and measures for knowing another contraceptive user were included, the coefficients on network composition remained consistent while the coefficient for knowing another contraceptive user was no longer significant (*results not shown)*. When partners were excluded from the definition of FP networks, the findings remained consistent though the magnitude of coefficients were smaller. Having a FP network was associated with 1.2 times higher use of contraception (95% CI, 1.04–1.38; *p* ≤ 0.01). While the coefficient on provider-only FP networks was no longer significant, the coefficients on social-only FP networks and mixed FP networks remained significant. The estimated e-value for the RR of having a FP network was 3.2, meaning that an unmeasured confounder would need to have a RR greater than 3.2 associated with both couples’ contraceptive use and whether men have a FP network in order to explain away the main results. The estimated e-values for social-only network, provider-only network, and mixed FP network were 3.6, 3.0, and 4.1.

## Discussion

There exists very little evidence examining men’s networks for information and advice about FP and how these networks are associated with women’s contraceptive use. The findings from this novel egocentric study show that many men (82%) report having at least one person who influences their FP use by providing information, advice or guidance. Although men are twice as likely to be influenced by providers by relying on them for information, advice and guidance about FP (54%) compared to family and friends (26%), men having any kind of FP network were 2 times more likely to report their partner using modern contraception compared to men with no FP network. Having more than one tie was not differentially associated with contraceptive use compared to having just one tie. Overall, the findings demonstrate that having at least one tie who influenced their FP use, be it a social relationship or a provider relationship, is what differentiates men who report that their partner is using contraception. Interventions to ensure women can exercise their reproductive autonomy and use contraception if desired should adopt a multi-pronged approach that includes male partners and their networks to foster their supportive engagement in contraceptive decision-making.

Men who reported being influenced by their partner for FP decision-making, by turning to them for FP information, advice and guidance, are even more likely to use contraception. This finding is consistent with evidence from Uganda where married men were less likely to rely on health providers or their peers, and they preferred to rely on their partners due to their partners’ experience with side effects, knowledge gained from their own peers, and exposure to mass media campaigns and health providers [41]. Men who nominated their partner as their social tie influencing their FP decision-making likely have more open couples’ communication about contraception, a critical factor affecting contraceptive use [52, 53]. The absence of significant coefficients for joint couples’ roles in decision-making about contraception may mean that actual decision-making resides with the woman, but more work needs to be done to explore these mechanisms in depth. In addition, future research should also examine concordance within couples regarding perceptions of contraceptive use, since this study relied on men’s perceptions of their partner’s contraceptive use, which may differ from their actual contraceptive behaviors.

Relying on other social ties, specifically siblings, was also associated with higher contraceptive use as a couple, compared to relying on doctors, nurses and mid-wives. Similar to the role of women’s social ties in Madagascar which play a positive role in increasing the likelihood of contraceptive use [14], men’s social ties also play a supportive role for contraceptive decision-making. These results contrast with other research finding that, for women, they were more likely to receive negative and incorrect information about contraceptives through social ties [42] and that social networks can exert a negative influence on adoption of reproductive behaviors [43, 44]. Our findings are consistent with the network study in Ghana showing that couples’ contraceptive use was higher among men who received support through their social networks to use contraception [40]. Despite the positive influence of social ties, only half as many men turned to social ties compared to providers which could point to the more sensitive nature of this topic in these communities.

There were no differences in the likelihood of contraceptive use depending on the type of health provider. Men were just as likely to use contraception whether they relied on nurses, mid-wives or doctors for information and advice about FP, compared to when they relied on CHWs and health educators. While another study found that women in Madagascar were more likely to use contraception if they relied on CHWs in their FP network compared to relying on nurses, mid-wives and doctors [14], the association in this study was not statistically significant for men in this study. The lack of precise estimation is possibly due to a small sample size. The findings also demonstrate that men primarily relied on women rather than men for FP information and advice. This tendency reflects the high proportion of women who occupy the roles of providers including mid-wives, nurses, CHWs and health educators tend to be women, in addition to partners and female siblings.

Similar to past work [8], men in this study were more likely to use contraception if they knew someone else using contraception. Yet, the associations were attenuated when network composition was included. Having FP network that is supportive of contraceptive use may be more important than having a network that includes contraceptive users [6, 8, 9, 11, 12]. Valente et al. (1997) show that, among women, their perceptions of ties’ approval of contraceptive use was more important than their ties’ actual use [8]. While our data did not allow for a distinction between alters’ active use versus ego’s perception of their use, future research should explore the role of both actual and perceived contraceptive use by alters on contraceptive use. Specifically, socio-network studies examining discordance in perceptions between egos and alters would be able to show whether perceptions of contraceptive use matter more than actual contraceptive behaviors. Research on social norms in sub-Saharan Africa has found that perceptions about whether friends or most people within a local reference group engage in a health-related behavior are potentially more important than peers’ actual behaviors [8, 13, 54, 55].

There are several study limitations. First, the relationship between having a FP network, including the composition of that network, and the decision to use contraception may be endogenous. For example, the reverse relationship could exist where couples decided to use contraception and men then selectively formed their FP network to validate that decision. Due to feasibility, it was not possible to conduct a complete network study to examine dynamic selection in this study population and establish the causal role of networks on contraceptive use. It is plausible that the main findings reflect a combination of selection (i.e., forming networks with individuals that have similar contraceptive behaviors to validates one’s contraceptive decisions) versus the influence of those networks on contraceptive behaviors. As with other egocentric network studies, this could not be teased out with the data. For this reason, we consistently referred to associations rather than using causal language. If it was possible to distinguish between selection and influence of networks, our estimates are likely to be smaller in magnitude though not zero, as put forth by Fowler and Christakis (2008) [56]. In addition, the opportunity for men to selectively form their FP network may be limited in this particular study context. Half of the men had a provider-only network and were constrained in selectively forming their FP network. In the study area, there are typically only 1 health educator or CHW per village and 1 nurse at the public health center providing FP. Men may have more ability to selectively form their social-only network. Future research using multiple data waves would facilitate identifying the causal influence of FP network composition on contraceptive use. Furthermore, if there was an omitted variable which influenced couples’ decision to use contraception (e.g. men’s personal preference for contraceptive use) and this factor also influenced FP network formation, the relationship between that omitted variable and these two variables would need to have a RR of at least 3.2, based on the e-value sensitivity analyses proposed by VanderWeele and Ding (2017) and Oster (2019) [50, 51], to completely explain away our main findings. The example of another unobservable (access to health services) did not meet this threshold for ruling out the main findings.

Second, men’s networks may be closely tied to their female partner’s social networks, by virtue of being in a couple. One of the study’s limitations is that the data were not collected by couple, therefore it is not possible to assess correlations in a couples’ FP networks. The results could be confounded by the female partner’s FP network which we cannot adjust for. Due to potential sensitivities of interviewing couples about FP and sexual health/practices in Madagascar, it was more feasible to recruit men without their partner. There is no study to our knowledge comparing differences by FP networks within couples and future studies examining couples would be beneficial. Third, there may be concern that including female partners within men’s networks is problematic because the outcome is simultaneously measuring those female partners’ contraceptive behaviors. Yet, when female partners were excluded from the definition of men’s FP networks, the results remained consistent.

Fourth, it is not possible to rule out the influence of potential unmeasured confounders such as shared environmental factors that may influence both men’s FP networks and contraceptive use. One such unmeasured factor might be access to FP services. The analyses adjusted for differences by fokontany (sub-district) which were statistically significant and suggest that there may exist differences in ease of access to FP services. Whether men have a FP network and the composition of that network may vary with their access to FP providers, thereby potentially biasing our estimates away from the null. However, there is no known research demonstrating such an association, and the participants in our study were all equidistant from FP providers since they were recruited from the same geographic area. One study from Madagascar showed that integrating FP services at the community level to improve access resulted in a two-fold increase in contraceptive use [57], but the effect size estimated from that study is smaller than our estimated e-value of 3.2. Therefore we believe it is unlikely that failure to account for confounding by access to FP services could completely explain away the main findings. Fifth, as an egocentric study, the data did not include alter-level contraceptive use for comparison to ego’s perceptions. Sixth, the sample may not be representative of rural populations in Madagascar since the villages were within a 5 km radius of a well-resourced semi-urban town. Couples’ contraceptive use in the sample (66%) was higher than reported contraceptive use (36%) in Madagascar as a whole [45], potentially due to ease of access to providers in the study region. Lastly, while the difference in missingness of covariates just misses statistical significance at the 5% level, it suggest that the analysis likely provides conservative estimates of the relationship between FP network and contraceptive use because the men without a FP network who were not included in some of the analyses may have been more difficult to engage with the survey and thus had missing data and biased the results towards the null.

## Conclusions

Whether men have a FP network distinguishes if couples are using contraception. Men vary in terms of whether they relied on providers or social ties for FP information and advice. Yet all men who had a FP network were significantly more likely to use contraception as a couple compared to those with no FP network. While men are more likely to rely on providers than social ties, these social ties can be just as important in providing support for contraceptive decision-making. There is very limited research examining whether men’s networks influence women’s contraceptive use, even though existing research has shown that men can play an important role in either facilitating or impeding women’s contraceptive use [16–29]. This study broadens the examination of men’s roles by moving beyond a focus on individual-level characteristics, to examining men’s FP networks and their role in contraceptive decision-making. The study’s findings point to several important ways to foster men’s positive engagement in contraceptive decision-making. First, reaching men through providers continues to be important. There remains a critical need to foster couples’ open communication about contraception. Interventions that engage men’s broader social network, including friends and family members, are an untapped avenue for supporting women’s access to and use of contraception in pursuit of universal access for all women.

## Data Availability

The datasets used and/or analyzed during the current study are available from the corresponding author on reasonable request.

## Appendix

**Table 5:** Association between network characteristics and couples’ use of contraception adjusting for couples’ pregnancy desires and contraceptive decision-making as a couple

	Dependent variable: Couple currently using modern contraception		
	(1)	(2)	(3) *Excludes partners from network variables*
*Independent variables*			
Composition of FP network			
No FP network *(Reference group)*	–	–	–
Social-only FP network	2.33^c^	1.55^b^	1.27^b^
	(0.43)	(0.24)	(0.10)
Provider-only FP network	1.91^c^	1.58^a^	1.17
	(0.27)	(0.34)	(0.11)
Mixed FP network	2.54^c^	1.74^a^	1.46^c^
	(0.40)	(0.44)	(0.14)
**Couples’ pregnancy desires**			
Wants to become pregnant *(Reference group)*	–		
Does not want to become pregnant	1.57e+ 06^c^		
	(1.68e+ 06)		
Is not certain	8.38e+ 05^c^		
	(8.70e+ 05)		
**Decision-making about contraceptive use with partner**			
Man decides *(Reference group)*		–	
		1.56	
His partner decides		(0.63)	
		1.22	
Couple decides together		(0.47)	
Observations	145	132	164
Mean of dependent variable	0.68	0.76	0.65

^a^ significant at 0.05; ^b^ significant at 0.01; ^c^ significant at 0.001

Note: We used generalized linear model with Poisson regression and robust standard errors. We reported relative risk ratios. Each column presents the findings of a separate regression model. Covariates included in each of these 4 regression models were age, household size, marital status, primary school completion, and household earnings. We also included sub-district fixed effects. We used cluster robust standard errors at the sub-district level. Social-only FP network included social ties such as partner, friend, and other family members. Provider-only FP network included provider ties such as CHWs, health educators, nurses, mid-wives, and doctors. A mixed FP network included both social ties and providers ties

## Abbreviations

CHW: Community health worker
FP: Family planning
IUD: Intra-uterine device
RR: Relative risk

## Footnotes

1 In the survey, we asked about family planning networks using the term *fanabeazana-aizana* which refers to family planning generally and includes both modern and traditional family planning methods. When asking about alters’ support of contraceptive use, we specifically asked about modern contraceptive use (“*Fomba fanabeazana-aizana maoderina”*).

2 On April 10, 2020: 1 USD = 3750 Malagasy Ariary.
